# Mismatch repair deficiency associated with complete remission to combination programmed cell death ligand immune therapy in a patient with sporadic urothelial carcinoma: immunotheranostic considerations

**DOI:** 10.1186/s40425-015-0104-y

**Published:** 2015-12-15

**Authors:** Michael P. Castro, Neal Goldstein

**Affiliations:** Personalized Cancer Medicine, PLLC, Queen’s Physician Office Building II, 1329 Lusitana St. Suite 609, Honolulu, HI 96813 USA; Clarient GE Inc, 31 Columbia, Aliso Viejo, CA 92656 USA

**Keywords:** Mismatch repair deficiency, Microsatellite instability, Immunotherapy, Urothelial cancer, PD-L1, PD1-L2, MSH2, MSH6, Hypermutator, Mutation load, Immunotheranostic, Precision immunology

## Abstract

**Background:**

Mismatch repair deficiency (MMRD) is a common pathway of malignant transformation accounting for approximately 15–20 % of human carcinogensis. It has been postulated that MMRD increases tumor antigenicity and highlights a role for immunotherapeutic approach MMR-deficient cancers. This strategy was pursued in a patient with upper tract urothelial carcinoma, and the results are reported here.

**Case Presentation:**

Molecular profiling was performed using next generation DNA sequencing and (IHC) testing for MMR and PD-L1. A patient with sporadic, high grade urothelial carcinoma of the renal pelvis was found to have a hypermutator genotype with 73 mutations occurring amidst 62 known drivers of malignancy, and 340 VUS alterations. MMR deficiency phenotype was confirmed by the absence of MSH2 and MSH6 as well as deleterious mutations in these genes. IHC staining for programmed cell death ligand-1 [PD-L1] revealed 2+ staining in 80 % of cells. The patient gained access to combination immunotherapy trial utilizing MEDI4736 and MEDI0680 through a clinical trial. The patient achieved a prolonged, complete remission within two months and had no severe ill effects from the treatment.

**Conclusion:**

Given their ability to generate neo-antigens, MMR-deficient cancers may be uniquely susceptible to immune checkpoint inhibitor strategies, including urothelial tract cancers. Screening for MMR deficient cancers has the potential to become a routine strategy for evaluating the role of PD-L1 inhibitors for patient with advanced disease. (Trial registration: Clinicaltrials.gov NCT00938834. Registered 13 July 2009)

## Background

The integrity of DNA replication depends on intact mismatch repair [MMR] genes. Patients with epigenetic silencing or deleterious mutations involving these key enzymes have a predilection for a variety of cancers due to altered microsatellite nucleotides and replication errors causing a hypermutant phenotype with hundreds or thousands of mutations. These normally highly conserved areas of the genome are comprised of repetitive nucleotide sequences found in both exonic and intronic DNA. In contrast to chromosomal instability, microsatellite instability (MSI) caused by MMR deficiency represents a distinct pathway of carcinogenesis through mutations in genes controlling growth pathways.

The hereditary syndromes involving mutations of mismatch repair enzymes (MLH1, MSH2, MSH6, and PMS2) were originally identified by Dr. Henry Lynch and is now divided into Lynch syndrome I consisting of colon cancer-only families, and Lynch syndrome II that includes a variety of malignancies such as genitourinary, gynecologic, and other gastrointestinal cancers [1]. Deleterious mutations in MSH2 are specifically associated with both upper (5.6 %) and lower tract (12.3 %) urothelial cancers [2]. However not all patients with urothelial carcinoma possessing MSI have the hereditary syndrome, and somatic knockout of the MMR genes is possible without any family history of cancer: MSI can be identified in approximately 3 % of bladder cancers and 15 % of sporadic upper tract urothelial malignancies [3, 4].

Paradoxically, MSI-derived cancers have a better prognosis than microsatellite stable (MSS) cancer in early stage, but are more poorly responsive to chemotherapy in the metastatic setting. In colorectal cancer, MSI confers a superior prognosis, adjuvant fluoropyrimidine therapy produces no benefit and may lead to inferior survival among patients with stage II disease. But in advanced colorectal cancer, MSI is frequently associated with BRAF mutations and portends an unusually poor prognosis. In urologic cancers as well, MSI indicates a better prognosis for early stage cancers [5]. On the other hand, even in germ cell cancer, the most chemotherapy-responsive solid tumor, MSI is associated with chemotherapy resistance [6].

MSI-derived cancer appears to be more antigenic than MSS malignancies and has a special susceptibility to immunotherapeutic strategies. Among patients with MSI-derived colorectal cancer, the response rate, duration of response, progression free and overall survival after treatment with an immune checkpoint PD-1 inhibitor, pembrolizumab, were vastly superior to the microsatellite proficient cancers [7]. While this landmark trial contained a group of patients with non-colorectal cancer, patients with urothelial cancer were not included in the study. Thus we present here the complementary experience in a patient with metastatic, mismatch repair deficient (MMRD) urothelial carcinoma of the renal pelvis. PD-L1 expression occurs in 45 % of urothelial cancers [8] and has a 50 % response rate to PD-L1 inhibitor therapy [9]. MEDI4736 is a human IgG1 monoclonal antibody that binds specifically to PD-L1 and demonstrated durable antitumor activity during phase I/II testing [10]. MEDI0680 is a humanized IgG4 mAb that blocks PD-1, thus interfering with ligand binding of both PD-L1 and PDL-2. This patient enrolled on a phase I clinical trial testing the combination of MEDI4736 and MEDI0680 [11].

## Case presentation

The patient is a 45-year old woman of Japanese descent with urothelial carcinoma of the right renal pelvis. The family history was negative for any malignancies in first-degree relatives. She has been a life-long non-smoker and has no occupational exposure to aniline dyes, radiation, or other chemicals. After presenting with gross hematuria in November 2013, a CT-IVP showed an abnormal, mass-like infiltration measuring 2.9 x 2.4 x 3 cm of the middle to lower right renal collecting system involving the renal parenchyma and invading the sinus fat. Ureteroscopy and biopsy revealed a transitional cell malignancy of the right lower pole renal calyx. In December 2013, she underwent a hand-assisted right nephroureterectomy.

**Fig. 1:**
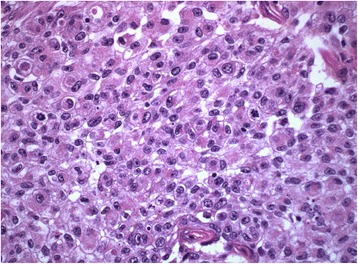
H&E high power showing high-grade urothelial carcinoma

Pathology revealed high-grade urothelial carcinoma invading the renal parenchyma and peripelvic fat without a significant inflammatory component (Fig. 1). The resection margins were negative. She was deemed as having G3 pT3N0M0, AJCC Stage III cancer. Post-operatively, she received 6 cycles of adjuvant gemcitabine-cisplatin chemotherapy and completed the treatment by July 2014. A CT scan immediately following adjuvant treatment revealed a new contralateral lymphadenopathy measuring 1.2 cm. Subsequently, a repeat scan revealed a new nodular lesion in the right renal bed and an increase in the dimensions of the left sided lymph node to 2.3 cm including two additional areas of metastatic disease: a lesion overlying the right iliopsoas muscle and the left para-aortic adenopathy. PET scanning disclosed that the 3 lesions seen on CT were hypermetabolic. A CT-guided biopsy confirmed metastatic urothelial carcinoma.

Molecular profiling was performed at Foundation Medicine, Inc. utilizing next generation DNA sequencing to identify actionable genomic alterations in key oncogenes and tumor suppressor genes [exonic regions of 315 genes]. This testing revealed 73 mutations among 62 genes (Table 1). Additionally, 340 variants of unknown significance [VUS] abnormalities were also identified among 166 genes (not shown). Three deleterious mutations were identified in MSH2 (A913fs*2, E226*, E580*) and one in MSH6 (R361H) (Table 1).

**Table 1:** Molecular profiling results from FoundationOne™ testing. 73 deleterious mutations were identified among 62 genes known drivers of cancer. Additional 340 variants of unknown significance [VUS] abnormalities were identified among 166 genes (not shown). Four mutations were identified in mismatch repair genes MSH2 and MSH6

ERBB4	R842Q	TP53	P222L, R175C, R282W	MSH6	R361H
FBXW7	R689Q	APC	R856C	MSH2	A913fs^*^2,E580^*^ E226^*^
FGFR3	R248C	AR	R616H		
JAK2	D319N	ARID1A	R1989^*^	MYST3	R79Q
KRAS	A18T	ARID2	L390fs^*^1, Q819^*^, R1677^*^	NOTCH2	R2036^*^
NF2	R424C	ATRX	R2131, 6849 + 2 T > C	POLE	V411L
PTCH1	R135^*^	CHEK2	W97^*^	PRDM1	A5O2T
ROS1	A114OT	CIC	R1515H	PREX2	R1149H
TET2	E149^*^	CTCF	R11W	SLIT2	C1022^*^, R942^*^,2417+ 2 T>C
ATM	R1875^*^	CUL3	R148^*^		
ATR	R1082H	EPHB1	G642D	SMAD4	R361H
CDKN2A	p16INK4a, A68T, p14ARF l	FAT1	S4314fs^a^6	SMARCA4	R1093^a^
CHEK1	R160H	FLT3	485-1G > A	SPEN	R2081^a^
CREBBP	R2344W	GRIN2A	E1461K, R1206^a^	SPTA1	R374Q
EP300	R838C	HNF1A	S574N	TAF1	R1172^a^
FANCA	T1161M	KDM6A	R1279^a^	TGFBR2	R528C
NTRK1	R649W	MLL2	R4238C, R4904^a^	WT1	T358M

The hypermutant genotype was phenotypically evaluated with 4-gene MMR IHC testing that showed loss of expression of both MSH2 and MSH6 (Table 2).

**Table 2:** Mismatch repair IHC testing results

GENE	Antibody	Result
MLH1	G168-15	Normal expression
PMS2	A16-4	Normal expression.
MSH6	BC/44	Loss of expression.
MSH2	FE11	Loss of expression.

**Fig. 2:**
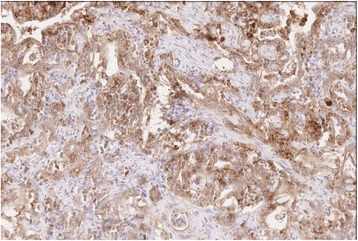
Strongly positive PD-L1 immunostaining (Clarient GE, Inc.) Sections were cut and dewaxed followed by application of the primary PD-L1 antibody (Cell Signaling Co., clone E1L3N, 1:150) and PD-1 antibody (CellMarque Co., clone MRQ-22, 1:300). After washes, DAB was used as the chromogen and slides were counterstained with hematoxylin

Germline DNA testing at Myriad Inc. was negative for all known Lynch mutations. Subsequently, the patient’s primary tumor was tested at Clarient GE, Inc. [Aliso Viejo, CA] for PD-L1 expression and found 2+ staining in 80 % of cells (Fig. 2)

.

**Fig. 3:**
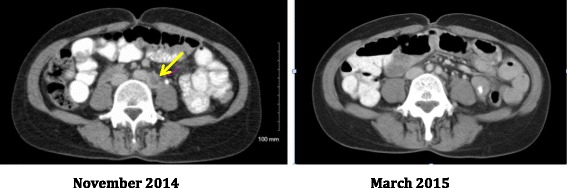
The baseline film in November reveals a necrotic lymph node in the left para-aortic region adjacent to the ureter. The follow-up study in March 2015 shows a complete disappearance of the lesion

The patient enrolled on a phase I clinical trial of an anti-PD-L1 inhibitor MEDI4736 and MEDI0680, [MedImmune Inc., Gaithersburg, MD] in Los Angeles [Clinical trial #: NCT02118337]. Within 2 months at the first radiographic evaluation, she had a complete disappearance of all cancer (Fig. 3). After 10 months of treatment, she developed grade 1–2 uritcarial rash and polyarthralgias in her hands and a positive ANA 1:160, but has no signs of severe or life-threatening autoimmunity or other criteria for a diagnosis of SLE. She has a confirmed continuous complete remission at 11 months and continues to participate on study.

## Conclusions

This patient had progressive, metastatic disease at the conclusion of platinum-based adjuvant therapy exemplifying the phenomenon of chemotherapy resistance and treatment failure characteristic of MMRD cancer. Despite the MSI phenotype, her primary cancer did not have a brisk tumor infiltrating lymphocyte (TIL) population. In retrospect, this observation is consistent with anergy caused by a strongly PD-L1 expressing cancer, and hinted at her inevitable relapse rather than the favorable outcome usually associated with MSI-derived cancers. At relapse, the molecular profiling evaluation revealed a hypermutant genotype and led to discovering the somatic knockout of MSH2 and MSH6 induced by deleterious mutations. While this patient’s cancer proved to have deleterious mutations in MSH2 and MSH6, IHC testing for MSI to identify epigenetic silencing in the absence of mutation represents an exceptional example where NGS assessments of DNA cannot substitute for protein assessment.

Given the impracticality of inhibiting dozens of deranged driver pathways in this patient’s disease with any targeted or conventional cytotoxic strategy, the identification of MMRD and PD-L1 over-expression provided a plausible hypothesis for controlling her metastatic cancer, even though neither biomarker was required for participating on this clinical trial. Indeed, had those tests been negative, it would not have excluded an immunologic strategy. On the other hand, the identification of PD-L1 expression created enthusiasm for accessing checkpoint inhibitor therapy. The clinical trial she enrolled on is actively testing the combination of MEDI4736 mAb that blocks PD-L1 and MEDI0680 mAb that blocks PD-1, a dual blockade that may be more effective than targeting either target alone [12]. The results of this accruing trial are eagerly awaited.

To our knowledge, this is the first documented case of specifically characterized MMRD-urothelial cancer with a clinical benefit from PD-L1 immunotherapy. The significance of the observation derives from the universal experience of treating metastatic urothelial cancer where patients with platinum-refractory disease seldom have meaningful, durable responses to subsequent chemotherapy and usually survive no longer than a matter of months. Other PD-L1/PD-1 agents, MPDL3280A, atezolizumab, and MK3475, pembrolizumab, have already been reported as having impressive efficacy in urothelial cancer, but so far there has been no evaluation whether those patients’ cancers had MMRD [8, 13]. This case report begs the question of MMRD status in these studies to see if the association holds in a larger cohort.

Whole exome sequencing (WES) has demonstrated that mutation load has a direct bearing on a given cancer’s immunogenicity and predicts the likelihood of benefit from ipilumumab [14]. It follows that the MSI phenomenon which causes 10 to 1000 times more mutations than found in MSS cancers creates neo-antigens and increases antigenicity, thus explaining the usual inflammatory reaction of TIL, and accounting for the improved prognosis of early stage, MMRD cancers. At the same time, PD-L1 expression has emerged as one of the fundamental escape mechanisms by which malignant diseases bypass immunosurveillance resulting in metastatic progression and *adaptive immune resistance* [15, 16]. As such, PD-L1 testing in the primary tumor could emerge as a prognostic biomarker for relapse as well as a promising strategy for selecting patients most likely to benefit from adjuvant checkpoint inhibition. It is also noteworthy that while the NGS analysis in this report contained only 315 genes, the 131 % frequency of genetic alterations suggests that more limited assays may be able to substitute for WES for determining elevated mutation load as a biomarker for immunotherapy in the future.

MMRD has been identified as a predictive biomarker for PD-1 immunotherapy in patients with colorectal cancer leading to unsurpassed response rates, 90 % disease control, and prolonged survival. The results in other non-colorectal, MSI-derived cancers are just as impressive in that study [7]. At the same time, MSI may represent only one of a variety of pathways for generating increased mutation load, enhancing antigenicity, and creating a high chance for benefit from immunotherapy. The susceptibility of heavy carcinogen-associated cancers, such as lung and head and neck cancers, to checkpoint immunotherapy supports the notion that the antigenicity of cancer increases with rising mutation burden from any cause. Emerging data in hepatoma and head and neck cancer also suggests that malignancies caused by viral infections capable of altering normal cellular antigen expression, including HBV, HCV, EBV, and HPV might also be uniquely responsive to immune checkpoint inhibitors [17, 18].

At the present time, the optimal biomarker approach for identifying benefit from immune checkpoint-directed therapy remains an open and complex issue. The clinical role of PD-L1 biomarker testing while not quite “investigational” is still in a formative stage. Already, it has been demonstrated that PD-L1/PD-1 IHC staining intensity is correlated with the likelihood and duration of benefit in lung cancer. On the other hand, the absence of staining does not preclude the possibility that immune checkpoint inhibition will lead to disease control [19, 20]. The ideal antibody has not been identified, such that the commercially available PD-L1 antibodies have specificity for different epitopes and can yield discordant results from the same tissue [21]. Additionally, discordant expression between a primary tumor and its metastases as well as heterogenous staining, ranging from 0 to 3+ within a given tissue sample, argue for a skeptical interpretation of any negative IHC result. Quantitative PCR testing of PD-L1 appears promising [22], but so far has not been prospectively evaluated against IHC techniques. Finally, PD-L1 staining by itself is no guarantee that targeting this mechanism will control the disease. Other mechanisms modulate the immune response, including IDO-1, PD-L2 and LAG-3, and represent additional potential biomarkers of resistance [23]. Even a desmoplasic tumor microenvironment arising from expression of fibroblast activated proteins that impede T cells from gaining access to cancer cells appears to facilitate immune tolerance, and could represent a marker of resistance as well as another mechanism for drug development to overcome [24]. On the other hand, the absolute lymphocyte count and its incremental changes during treatment appear to be robust predictors of survival benefit among melanoma patients treated with ipilumumab [25].

Despite the daunting complexity that emerges from these discoveries, they offer hope that assay-directed, rational targeting of key mechanisms of immune evasion, i.e. “precision immunology,” will become feasible and direct the optimal combination strategies to overcome resistant disease for individual patients. At the moment, both MMRD and PD-L1 appear to have merits as biomarkers for PD-L1/PD-1 antibodies with regard to using a one-sided predictive rule, making immunotherapy a priority whenever either one or both are present. Also promising, ICOS [26] and NY-ESO-1 [27, 28] represent candidate markers for predicting response to CTLA-4 antibodies. The 58–60 % decrease in disease progression associated with combination immune checkpoint inhibitor therapy for untreated melanoma creates a high priority for identifying which patients need the combination and its attendant risks, and which can benefit from PD-L1 therapy alone and spared unnecessary cost and toxicity [29, 30].

While the new biomarker science of immunodiagnostics matures, an increased and broadly applied index of suspicion for detecting MMRD among the entire spectrum of malignant disease [especially GI, GU, and GYN cancer] appears warranted and has important *immunotheranostic* implications. Drug development targeting either MMRD or PD-L1 irrespective of tumor site of origin could well turn out to be an effective strategy for advancing patient outcomes compared to conventional, disease site-limited trial design and the cytotoxic options that perform poorly in MMRD cancers. With appropriate regulatory support, the success of such trials promises to define a new paradigm in the treatment of human cancer.

## Trial registration

Clinicaltrials.gov NCT00938834. Registered 13 July 2009.

## Patient consent

The patient provided signed written consent for the case report and the images provided herein to be published.

## Abbreviations

IHC: Immunohistochemical
MLH1: Mut homolog 1
MSH2: MutS protein homolog 2
MSH6: MutS protein homolog 6
PMS2: Mismatch repair endonuclease PMS2
mAb: Monoclonal antibody
MMR: Mismatch repair
MMRD: Mismatch repair deficiency
MSI: Microsatellite instability
MSS: Microsatellite stable
PD-L1: Programmed cell death ligand-1
SLE: Systemic lupus erythermatosis
AJCC: American Joint Commision on Cancer
HBV: Hepatitis B virus
HCV: Hepatitis C virus
EBV: Epstein Barr Virus
HPV: Human papilloma virus
TKI: Tyrosine kinase inhibitor
LAG3: Lymphocyte-activation gene 3
ICOS: Inducible T-cell COStimulator
CTLA-4: Cytotoxic T-lymphocyte-associated protein 4
PET: Positron emission tomography
CT-IVP: Computed tomographic intravenous pyelogram. DNA, deoxy-ribonucleic acid
BRAF: Serine/threonine-protein kinase B-Raf. WES, Whole exome sequencing
